# Rapidly progressive locked-in syndrome secondary to atypical herpes simplex virus-1 rhombencephalitis in an immunocompromised individual

**DOI:** 10.1016/j.idcr.2024.e02027

**Published:** 2024-07-06

**Authors:** Jeffrey Xia, Rasha Ahmed

**Affiliations:** UCLA Medical Center, Los Angeles, CA, USA

**Keywords:** Herpes simplex virus, Encephalitis, Locked-in, Chronic lymphocytic leukemia, Magnetic resonance imaging, Neurology, Infectious disease

## Abstract

HSV-1 encephalitis (HSE) is the most common cause of fatal sporadic encephalitis in the United States. HSE in adults is most commonly due to the reactivation of HSV in the central nervous system (CNS) which results in CNS necrosis leading to neurological compromise. The most common symptoms include altered mentation, fever, seizures, and focal neurological deficits. HSE most commonly involves damage to the temporal lobes however can rarely involve other CNS structures such as the brainstem and cerebellum. Immunocompromised status may increase the risk of atypical HSE. HSE involvement of the brainstem, particularly the pons, most commonly cause neuro-ocular and neuro-bulbar deficits. Rarely can HSV brainstem encephalitis cause quadriplegia or locked-in syndrome. We present a case of HSV-1 rhombencephalitis complicated by locked-in syndrome in a patient with CLL.

## Introduction

HSV-1 is a double stranded DNA virus of the human Herpesviridae family and is a common infection with a global seroprevalence of 63.6 % [1]. HSV transmission occurs through direct contact of body surfaces containing viral contagion. HSV replicates in epithelial cells resulting in cell lysis and inflammation causing characteristic localized vesicular lesions [2]. HSV-1 tends to establish lifelong latency in the trigeminal ganglia, placing patients with latent HSV-1 at risk for reactivation particularly in times of immunocompromise [2].

HSV encephalitis (HSE) has an estimated incidence of approximately 1 in 100,000 to 150,000 individuals in the US [3]. Approximately 90 % of all HSE cases are due to HSV-1 [4]. The mortality rate of HSE ranges between 40–97 % if left untreated and 4–28 % if treated [4], [5]. Even when treated, HSE demonstrates high morbidity**:** in a multicenter observational study of 93 patients diagnosed with HSE, at one year from onset of HSE, 28 % died, 17 % had complete recovery, 23 % had mild disability, 19 % had moderate disability, and 13 % had severe disability [6]. The morbidity of HSE is thought to be likely secondary to neuronal necrosis extrapolated from mouse models [7]. While not completely known, both direct viral lysis of neuronal cells and collateral damage from immune response are thought to be the mechanism of the CNS damage in HSE, with more evidence supporting the latter [7]. Interestingly, HSV-1 encephalitis is not more common amongst the immunosuppressed and may entail either a more indolent course with minimal mild histopathologic changes on biopsy or more extensive brain damage despite limited signs of inflammation; However, inborn errors of immunity associated with HSE in children are currently being further investigated in adults which may offer more clarification [8], [9], [10].

Immunocompromised patients may present with HSE atypically. In an immunocompromised patient, presenting symptoms of HSE may be highly variable, ranging from absence of prodromal symptoms to a more rapid onset of symptoms requiring hospitalization compared to immunocompetent hosts [5]. Here we present a case of an immunocompromised patient presenting with prodromal symptoms of HSE with atypical CNS involvement resulting in HSV-1 rhombencephalitis complicated by locked-in syndrome.

## Case presentation

A 61-year-old man with chronic lymphocytic leukemia (CLL), dyslipidemia, type 2 diabetes, and hypertension presented to our hospital with subacute onset of fatigue, confusion, and unsteady gait. Ten years prior to this admission, he was diagnosed with Rai stage 3 CLL with poor prognostic markers including positivity for ZAP40 and CD38, unmutated IGVH, and del 17p. He was treated with alemtuzumab and methylprednisolone achieving remission. Five years before this admission, he was hospitalized for severe diabetic ketoacidosis. Two years before this admission, he was hospitalized for COVID-19 and found in CLL relapse, later treated with acalabrutinib achieving remission again; otherwise, he had no history of recurrent infection or other infection requiring hospitalization.

Four days prior to admission, he traveled by airplane and upon arrival, friends noted he was fatigued, confused, slightly disheveled, and ataxic. Collateral information from family members in his home state revealed that his symptoms started prior to his departure flight. These symptoms progressed until he was noted to have left-sided facial droop and was brought to our hospital for further evaluation. He was found to be alert but demonstrated delayed verbal responses with word finding difficulties. He was oriented to name and location only. He denied having recent fever, chills, nausea, vomiting, and diarrhea. His home medications were acalabrutinib, amlodipine, metformin, and hydrochlorothiazide which were all held for the entirety of his hospitalization. On presentation, he was afebrile with a blood pressure of 110/75 mmHg, heart rate of 99 beats per minute, respiratory rate of 18 breaths per minute, and an oxygen saturation of 96 % on room air. On physical exam, he demonstrated dysmetria on finger-to-nose testing and ataxia. Otherwise, all cranial nerves were intact. He had normal muscle bulk, normal muscle tone, no rigidity, no tremor, and 5/5 motor strength throughout. Sensation was intact throughout. Reflexes were normal throughout with the exception of absent bilateral Achilles tendon reflexes. The remainder of his physical exam was normal.

**Fig. 1:**
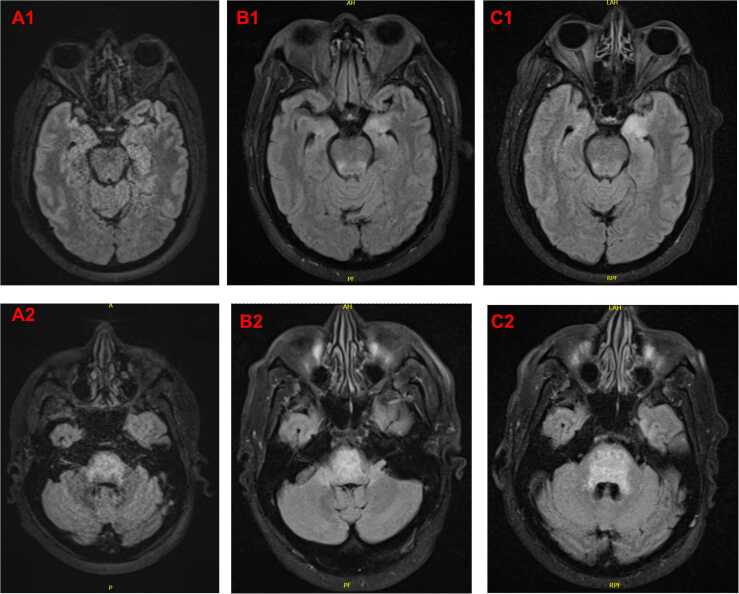
Magnetic resonance brain imaging of a patient with herpes simplex virus rhombencephalitis. Image set A are T2 sequence images obtained on hospital day 1. Image set B are FLAIR sequences obtained on hospital day 5. Image set C are FLAIR sequences obtained on hospital day 8. Images labeled with “1” are cuts through the hippocampi and images labeled with “2” are cuts through the pons at the infraorbital level.

On initial labs, he had a normal complete blood count, slight azotemia, and a hemoglobin A1c of 9.7 (Table 1). Serum lactate, TSH, high sensitivity troponin, and B-natriuretic peptide were all within normal limits (Table 1). He had an elevated erythrocyte sedimentation rate to 55 mm/hr, but C-reactive protein was not obtained during hospitalization (Table 1). An MRI/MRA brain with and without contrast was obtained which demonstrated multifocal amorphous, linear, and curvilinear enhancement and areas of diffusion restriction within the pons on T2/FLAIR; There were no mass-effect, hemorrhage, midline shift, hydrocephalus, and abnormal fluid collections; The cerebral and carotid arteries were normal (Fig. 1, A1–2). A lumbar puncture was obtained for cerebrospinal fluid (CSF) analysis which demonstrated 113 total nucleated cells comprised of 96 % lymphocytes and 4 % monocytes, along with 29 RBC/uL, glucose 187 mg/dL, total protein 57 mg/dL, and negative bacterial and fungal stain. He received only one lumbar puncture throughout his prolonged hospitalization.

**Table 1:** Admission serum labs.

Lab	Value	Reference
Sodium	138	135 - 146 mmol/L
Potassium	4.4	3.6 - 5.3 mmol/L
Chloride	101	96 - 106 mmol/L
Bicarbonate	21	20 - 30 mmol/L
BUN	23	7 - 22 mg/dL
Creatinine	1.34	0.60 - 1.30 mg/dL
Lactate	12	5 - 18 mg/dL
A1c	9.7	< 5.7 %
Calcium	9.6	8.6 - 10.4 mg/dL
Troponin	< 4	< 5 ng/L
BNP	40	< 100 pg/mL
TSH	1.6	0.3 - 4.7 mcIU/mL
WBC	6.53	4.16 - 9.95×10E3/uL
Hemoglobin	13.8	13.5 - 17.1 g/dL
Hematocrit	43.9	38.5 - 52.0 %
MCV	103.8	79.3 - 98.6 fL
Platelets	174	143 - 398×10E3/uL
Neutrophil count (%)	3.94 (60)	1.80 – 6.90×10E3/uL
Lymphocyte count (%)	1.92 (29)	1.30 – 3.40×10E3/uL
Eosinophil count (%)	0.0 (0)	0.00 – 0.50×10E3/uL
Basophil count (%)	0.01 (0.2)	0.00 – 0.10×10E3/uL
Erythrocyte sedimentation rate	55	≤ 12 mm/hr
IgM, serum	27	40 – 230 mg/dL
IgG, serum	617	700 – 1500 mg/dL
IgA, serum	< 7 mg/dL	74 – 426 mg/dL
IgE, serum	< 2 kIU/L	n/a
*Streptococcus pneumoniae* serotype 1 (IgG)	0.3	≥ 0.3 mcg/mL
*Streptococcus pneumoniae* 22 serotypes* (IgG)	< 0.3	≥ 0.3 mcg/mL
Rapid plasma reagin (RPR)	Negative	Negative
Borrelia burgdorferi Ab	Negative	Negative
Cocci IgM	< 0.150	< 0.150
Cocci IgG	< 0.150	< 0.150
Histoplasma antigen	Negative	Negative
HIV-1/2 Ag/Ab	Negative	Negative
Influenza A PCR	Negative	Negative
Influenza B PCR	Negative	Negative
COVID-19 PCR	Negative	Negative

* *Streptococcus pneumoniae* serotypes tested: 2, 3, 4, 5, 8, 9, 12, 14, 17, 19, 20, 22, 23, 26, 34, 43, 51, 54, 56, 57, 68, 70

He was initially managed with vancomycin, cefepime, ampicillin, and acyclovir (10 mg/kg IV every 8 h). Neurology was consulted, recommending 1 g/day methylprednisolone for possible chronic lymphocytic inflammation with pontine perivascular enhancement responsive to steroids (CLIPPERS) finding on MRI brain. On hospital day (HD) 2, his fatigue worsened. CSF studies were positive (cycle threshold not reported by lab) for herpes simplex virus-1 (HSV-1) and negative for all other pathogens and conditions tested on the CSF meningoencephalitis and autoimmune/paraneoplastic panels (Table 2). With infectious disease consultation, treatment was adjusted to acyclovir monotherapy to manage atypical HSV-1 rhombencephalitis. On HD 3, his clinical status continued to deteriorate with worsening encephalopathy. He developed dysarthria and was no longer able to hold intelligible vocalization but followed simple commands. He started to aspirate and was assessed by speech language and pathology and was found to have absent versus delayed laryngeal response reflex. On HD 5, he developed diplopia with new left cranial nerve six palsy. He also developed new right cranial nerve seven palsy. MRI brain was repeated which demonstrated new FLAIR hyperintensity and restricted diffusion of left greater than right hippocampi; Previously demonstrated pontine enhancement was overall improved however there was interval increased involvement of the middle cerebellar peduncles and cerebellar flocculus; There were also few new foci of pontine petechial hemorrhage (Fig. 1, B1–2). On HD 6, he developed weakness of the right extremities (3/5 motor strength of the upper extremity, 2/5 strength of the lower extremity) with normal strength of the left extremities. At this time, methylprednisolone was discontinued as any improvement would have been observed already for CLIPPERS. On HD 7, he was oriented only to name. His weakness progressed to 2/5 motor strength in all four extremities and required maximum assist in bed mobility. On HD 8, he was transferred to the ICU for acute hypoxic respiratory failure secondary to aspiration, intubated for airway protection as he was minimally responsive, and started on piperacillin-tazobactam for hospital acquired pneumonia later confirmed to be methicillin sensitive *Staphylococcus aureus* on tracheal aspirate culture with antibiotics narrowed to ampicillin-sulbactam. MRI brain was repeated which demonstrated progressive FLAIR hyperintensity and restricted diffusion in bilateral hippocampi and progressive FLAIR hyperintensity involving the pons, middle cerebral peduncles, and superior medulla (Fig. 1, C1–2). On HD 9, foscarnet was started for possible acyclovir-resistant HSV-1. On HD 16, he was found to have hypogammaglobulinemia; With immunology consultation, the hypogammaglobulinemia was thought to be secondary to active infection versus CLL, and he was provided 400 mg/kg intravenous immunoglobulins to be administered every 4 weeks until goal IgG > 700 mg/dL which was ultimately not achieved prior to his discharge (highest serum IgG achieved 617 mg/dL on HD 78). Mitogen/antigen induced lymphocyte proliferation panel, lymphocyte enumeration panel, tetanus and pneumonoccocal antibody titers, and whole exome sequencing of TLR3 gene were completed for further workup. Briefly, his T-cell subset counts were normal with reduced ratios (CD3 count 2107/cmm with 29 % ratio; CD4 count 1018/cmm with 14 % ratio), B-lymphocyte count and ratio were significantly elevated (>863/cmm with 63 % ratio), lacked response to tetanus and pneumococcal vaccines, and no clinically significant variants of TLR3 gene were identified.

**Table 2:** Cerebrospinal fluid studies.

Lab	Value	Reference
RBC	29	0 - 10 /uL
WBC	113	0 - 5 /uL
Lymphocyte (%)	96	40 - 80 %
Monocyte (%)	4	15 - 45 %
Glucose, CSF	187	43 - 73 mg/dL
Glucose, serum	313	65 – 99 mg/dL
Protein	57	15 - 45 mg/dL
Bacterial culture and gram stain	Negative	Negative
Fungal culture	Negative	Negative
*Escherichia coli* K1 PCR	Negative	Negative
*Haemophiles influenzae* PCR	Negative	Negative
*Listeria monocytogenes* PCR	Negative	Negative
*Neisseria meningitidis* PCR	Negative	Negative
*Streptococcus agalactiae* PCR	Negative	Negative
*Streptococcus pneumoniae* PCR	Negative	Negative
CMV PCR	Negative	Negative
HSV-1 PCR	**Positive**	Negative
HSV-2 PCR	Negative	Negative
HHV 6 PCR	Negative	Negative
Human parechovirus PCR	Negative	Negative
VZV PCR	Negative	Negative
Enterovirus PCR	Negative	Negative
*Cryptococcus neoformans/gatti* PCR	Negative	Negative
AMPA-R Ab CBA	Negative	Negative
Amphiphysin Ab	Negative	Negative
AGNA-1	Negative	Negative
ANNA-1	Negative	Negative
ANNA-2	Negative	Negative
ANNA-3	Negative	Negative
CASPR2-IgG CBA	Negative	Negative
CRMP-5-IgG	Negative	Negative
DPPX Ab IFA	Negative	Negative
GABA-B-R Ab CBA	Negative	Negative
GAD65 AB Assay	0.0 nmol/L	≤ 0.02 nmol/L
GFAP IFA	Negative	Negative
IgLON5 IFA	Negative	Negative
LGI1-IgG CBA	Negative	Negative
mGluR1 Ab IFA	Negative	Negative
Neurochondrin IFA	Negative	Negative
NIF IFA	Negative	Negative
NMDA-R Ab CBA	Negative	Negative
PCA-Tr	Negative	Negative
PCA-1	Negative	Negative
PCA-2	Negative	Negative
Septin-7 IFA	Negative	Negative

He could not be weaned off the ventilator and a tracheostomy was placed on HD 17, along with a gastric tube for continued tube feeds. On HD 19, he regained full consciousness off sedation. He regained no motor function, demonstrating complete flaccid paralysis from the head down. He communicated by vertical movements of his eyes only and demonstrated comprehension of speech and written language. Therefore, locked-in syndrome was suspected. Foscarnet was discontinued on HD 20 after lab resulted absence of acyclovir resistance. He completed 21 days of acyclovir on HD 23 and was transitioned to valacyclovir 500 mg twice daily for prevention of HSV. Repeat lumbar puncture was not performed after completion of HSV treatment. On HD 28, he had recurrent ventilator associated pneumonia with tracheal aspirate confirming *Klebsiella pneumoniae* and was treated with ceftriaxone. On HD 42, he was successfully transitioned off the ventilator and breathing with continuous trach collar. On HD 63, he developed recurrent hospital acquired pneumonia with tracheal aspirate confirming *Pseudomonas aeruginosa* and was treated with piperacillin-tazobactam. On HD 68, he developed a fever and was found to have fungemia with *Candida parapsilosis* despite lack of presence of intravascular catheters; The likely etiology of his fungemia was thought to be transient gut translocation secondary to extensive courses of antimicrobial therapy throughout hospital course. His echocardiogram and ophthalmologic exams were unremarkable, and he completed a 14-day course of intravenous micafungin. He was eventually discharged on HD 86 to long term care facility. His neurological exam did not improve since locked-in syndrome was initially suspected over 2 months prior to his discharge. He will follow up with his oncologist regarding continuing CLL treatment.

## Discussion

In our review of the literature, this case represents the first documented case of locked-in syndrome secondary to irreversible damage of the pons from atypical HSV-1 rhombencephalitis with survival after initial hospitalization. Our patient had four major immunocompromising factors including CLL, treatment with tyrosine kinase inhibitor therapy (acalabrutinib), secondary hypogammaglobulinemia, and uncontrolled type 2 diabetes. In particular from a pool-safety analysis involving 693 patients with identified pathogen, acalabrutinib is associated with any infection in 67 % of patients receiving this treatment, of which approximately 23 % had viral infection (n = 1 patient had Herpes simplex infection) [11]. Alemtuzumab treatment (monoclonal antibody targeting CD52 found on several immune cells including B lymphocytes) ten years prior to admission may have contributed to his immunocompromised state as a pooled-safety analysis discovered continued elevated risk of infection six years after alemtuzumab infusion in a cohort of multiple sclerosis patients, though highest infection risk was in the first two years post-treatment [12]. After thorough immunology workup including whole exome sequencing, his hypogammaglobulinemia was thought to be secondary to infection or CLL and unlikely to have contributed to his risk of severe HSE.

He presented with typical prodromal symptoms of HSE without overt signs of infection such as fever, leukocytosis, and significantly elevated ESR. However, he developed rapid neurological decline likely secondary to extensive central nervous system necrosis from HSE involving the pons and cerebellum in addition to bilateral temporal lobes. His HSE demonstrated a particular sequence of MRI brain findings, namely involvement of the pons discovered first, followed by involvement of hippocampi, cerebellar peduncles, and temporal lobes. Furthermore, the sequence of his clinical course somewhat contrasts the sequence of imaging findings, namely one of his initial presenting symptoms was ataxia representing cerebellar involvement and progressed to quadriplegia representing extensive anterior pons and medullar damage. Despite completion of early targeted treatment with acyclovir, he unfortunately developed locked-in syndrome likely secondary to extensive irreversible damage of the pons.

The typical presentation of HSE includes altered mental status, fever, seizure, and/or focal neurological deficits including ataxia, dysarthria, and hemiparesis [13]. The imaging study of choice is MRI brain with and without contrast which most frequently reveals hyperintense lesions on T2-weighted sequences involving the mesiotemporal and orbitofrontal lobes, demarcating areas of cerebral edema/inflammation. [14] When suspecting encephalitis, lumbar puncture should be completed for cerebrospinal fluid analysis; In HSE, the CSF most frequently demonstrates lymphocytic pleocytosis (100–400 cells/uL), elevated protein, elevated erythrocyte count, and PCR positivity of HSV DNA. [15].

It is known that immunocompromised individuals with HSE may present atypically, thus HSE along with other infectious encephalitides should be considered for acute encephalopathy in immunocompromised patients. In the largest known case series of 35 patients with HSE, only 28.6 % of immunocompromised patients presented with HSE prodromal symptoms [5]. Therefore, a high degree of suspicion should be held when considering encephalitis for immunocompromised patients as delays in diagnosis and treatment may result in worse outcomes. The mortality rate of HSE is approximately five times greater in immunocompromised patients compared to immunocompetent patients [5]. Furthermore, immunocompromised patients demonstrated more extra-temporal MRI brain abnormalities, with 25 % having brainstem involvement and 17 % with cerebellar involvement [5]. HSV brainstem encephalitis is a rare presentation of HSE with limited documentation in the literature. In a review of 24 patients with HSE involving the brainstem, most cases were infected with HSV-1 (79 %) and had involvement beyond the brainstem (71 %). Interestingly, only a minority of HSV brainstem encephalitis cases had immunocompromised status (20 %) [16]. Some of the most common neurological findings were neuro-ophthalmologic (81 %) such as impaired extraocular movements; altered mentation (50 %); ataxia (38 %). Quadriplegia was uncommon with three known cases documented (19 %) [16]. .

The treatment of HSE is 14 to 21 days of 10 mg/kg intravenous acyclovir every 8 h with dose adjustment for renal insufficiency. Acyclovir is converted by viral thymidine kinase to acyclovir monophosphate which inactivates viral DNA polymerase and inhibit viral replication. While treatment should be prioritized at the time of consideration of HSE diagnosis, studies have shown that two-thirds of patients with HSE develop significant long term neurological morbidity even with early treatment with acyclovir [17]. Resistance to acyclovir is rare in immunocompetent populations (0.3 %); Immunocompromised populations are at higher risk of resistance (4–7 %) [18]. Our patient was started on prophylactic valacyclovir, however there is currently no strong evidence of its efficacy in preventing recurrent HSE [19].

Lastly, the diagnosis of locked-in syndrome requires both retained cognitive ability and paralysis of oral structures and limbs [20]. The most common causes of locked-in syndrome are brainstem stroke and pontine hemorrhage; Rarer causes of locked-in syndrome include central pontine myelinolysis, brain stem tumors, and infection [20]. The localization of CNS damage to brainstem structures results in retained higher level, cerebral functions such as alertness, attention, and cognition. Locked-in syndrome secondary to HSE is exceedingly rare with one documented case in a 28-year-old immunocompetent patient who progressed to locked-in syndrome one week after symptom onset; However, he further progressed into a comatose state within five days of admission, later expiring eleven days after admission due to bronchopneumonia [21].

## Conclusion

Herpes simplex encephalitis (HSE) frequently presents with a prodrome of altered mental status, fever, and/or focal neurological changes. Early recognition and management of HSE is critical as HSE has high mortality if left untreated. A high degree of suspicion should be practiced for immunocompromised patients who may present with HSE atypically. Even with appropriate treatment, HSE has high morbidity and may result in significant disability.

## Ethical statement

All procedures were performed in compliance with relevant laws and institutional guidelines and have been approved by the appropriate institutional committees.

## CRediT authorship contribution statement

**Jeffrey Xia:** Writing – original draft, Conceptualization. **Rasha Ahmed:** Writing – review & editing, Conceptualization.

## Declaration of Competing Interest

The authors declare that they have no known competing financial interests or personal relationships that could have appeared to influence the work reported in this paper.
